# Contracted Aorta

**Published:** 1829-03

**Authors:** 


### CONTRACTED AORTA.

### *Case in which the Aorta was nearly Impervious.*

(La Charité.)

Several instances of remarkable contraction of the aorta are mentioned by medical writers, and two cases have been recorded in which its canal was quite obliterated. One of these is detailed by Graham, of Glasgow; the other was inserted in the 33d volume of Corvisart's Journal.

A shoemaker, ninety-two years old, was admitted into La Charité, June 19th, 1827. He was short, rather thin, and his head small. His intellect was too weak to enable him to give a satisfactory account of his former state of health: however, he affirmed that the right side of his mouth, and the corresponding arm, had been paralysed. When relating this he could use both arms equally well; but the right appeared slightly bent, as in adduction. His head was habitually bald; the temporal arteries beat powerfully; the pulse was hard, frequent, and regular; the skin hot and dry; the tongue dry and horny; notwithstanding which, he was constantly asking for food. He was at first constipated, and after some time very relaxed. Nothing peculiar could be discovered in his respiratory organs, saving that percussion below the right produced a more obscure sound than below the left clavicle.

From constantly lying on the back, the integuments of the most prominent part of the sacrum ulcerated; and the patient, after a protracted abode in the hospital, gradually sunk, without presenting any thing else worthy of notice.

His body was opened four-and-twenty hours after death A small quantity of opaque serosity was found between the arachnoid membrane and pia mater, which were, however, removed with great facility. Both the hemispheres of the brain, and particularly the left, exhibited numerous traces of former apoplectic extravasations of small extent, and of which some were deep-seated and others situated superficially. The most considerable extravasation was in the substance of the left corpus striatum. An alteration which was observed in several spots of the exterior of the brain presented these characters: The gray substance was wanting to a greater or less extent, while the arachnoid membrane corresponding to it was entire. Underneath this membrane was a superficial erosion, at the bottom of which was a thread-like cellular membrane, yellow as ochre, and injected with a small quantity of milky serum. The small depressions indicating the destruction of the cineritious substance were either lined by a yellow cellular membrane, injected with serosity of the same colour, or by a smooth transparent membrane containing a thin serosity. The other parts of the brain exhibited no unusual appearance.

The heart was of the ordinary size. The internal membrane of the left ventricle was somewhat thickened, and in colour resembled milk. The sigmoid valves were in several parts incrusted with an osteo-calcareous matter.

The aorta, at its origin of about its usual circumference, very soon gave off the arteria innominata, the caliber of which was much larger than natural. After furnishing this branch, the aorta became much smaller, took an oblique direction upwards and towards the left carotid, which it gave off, and then, bending itself backwards at an acute angle, descended, exhibiting a slight enlargement. It now gave off the left subclavian, which was also much enlarged at its origin, and speedily took a perpendicular direction, and was sensibly smaller before any branch came off from it. The aorta forthwith presented a most conspicuous circular contraction, and very similar to that which a tight ligature would produce; then resuming its proper size, it had a slight dilatation, which produced an evident curvature towards the left side. The caliber of the aorta, in its course down the abdomen, appeared unnaturally small, particularly towards the lower part; and the size of the external iliacs was not in proportion to the bulk of the inferior extremities.

At the termination of the right subclavian, which was much augmented in size, several large arteries were given off. The transversalis scapulæ and cervicalis posterior, each nearly as large as the humoral artery, ran in the usual direction, but were remarkable for the thickness of their coats and their numerous sinuosities. The first preserved its caliber undiminished, until it arrived at the angles of the fourth and fifth ribs, between which it penetrated, furnished their anterior and posterior intercostals, crept along under the pleura for a short space, and, forming a continuation with an intercostal artery, terminated in the aorta, about half an inch above its contraction.

The cervicalis posterior, taking a more limited course, and descending more directly along the upper and hinder part of the back, was divided into three large branches, which, after separately penetrating into the thorax between the first four intercostal spaces, and supplying the corresponding ribs with their respective arteries, reached the aorta, into which they poured their contents through three large orifices.

Similar peculiarities were observed on the left side. The transversalis scapulæ and the cervicalis posterior, somewhat smaller; but taking the same course, penetrated into the chest likewise, and terminated in the left side of the aorta, below the contracted part. On this side it was also remarked that the superior intercostal, arising from the subclavian, communicated with the second aortic intercostal.

The right and left internal mammeries were very large, their diameters exceeding that of the humeral. Both of them, after running their usual course, and becoming a little narrower towards the inferior part of the thorax, again increased considerably in size, became very tortuous; then uniting with the epigastric, and forming with it a common trunk, surpassing in magnitude that of the external iliacs, terminated in the crural artery, whose diameter it considerably augmented.

The coats of the aorta presented no trace of diseased structure barring a few insulated spots where they appeared to be slightly thickened. Near the contracted part, too, the coats seemed to be equally sound. The stricture had the form of a regular circle interiorly, and the diameter of its bore was no larger than a crow-quill.

